# Pupil response as an indicator of hazard perception during simulator driving

**DOI:** 10.16910/jemr.10.4.3

**Published:** 2017-11-06

**Authors:** Florentin Vintila, Thomas C. Kübler, Enkelejda Kasneci

**Affiliations:** University of Tübingen, Germany

**Keywords:** Driving, Stress indicators, Pupil diameter, Attention, Visual field defect, Wavelets, Supervised classification

## Abstract

We investigate the pupil response to hazard perception during driving simulation. Complementary to gaze movement and physiological stress indicators, pupil size changes can provide valuable information on traffic hazard perception with a relatively low temporal delay. We tackle the challenge of identifying those pupil dilation events associated with hazardous events from a noisy signal by a combination of wavelet transformation and machine learning. Therefore, we use features of the wavelet components as training data of a support vector machine. We further demonstrate how to utilize the method for the analysis of actual hazard perception and how it may differ from the behavioral driving response.

## Introduction

The size of the human pupil regulates to the amount of light that enters the eye. An increase in luminance therefore results in a fast constriction of the pupil, a luminance decrease in a gradual ‘unconstriction’. The pupillary light reflex (PLR) ( 1 )regulates the light influx. On the other hand, there is a well-studied correlation between pupillary dilation and cognitive factors such as workload( 2 ), surprise( 3 ), attention( 4 ), and emotional arousal( 5 ).

Pupil dilation constitutes a proxy for indirect measurement of these cognitive factors, which would otherwise only be visible with costly and intrusive measurements such as EEG. Through behavioral observation, however, one can only measure the superposition of PLR and cognitive influences. Both, ‘unconstriction’ due to a luminance change and pupil dilation due to an increased arousal level result in a larger pupil. This fact effectively limits the usefulness of pupil dilation in most practical applications, as an equiluminant surrounding is only realistic for laboratory experiments.

 Not only are the causes of PLR unconstriction and cognitive pupil dilation different, but also different brain regions trigger them and they manifest through different components of the eye musculature. PLR is driven mainly by the constriction and relaxation of the iris sphincter muscle; cognitive changes innervate the iris dilator muscle( 1 ),( 6 ).

 With their work on the ‘index of cognitive activity’ (ICA) Marshall demonstrated that these processes are in fact so different in their manifestation (especially in the speed and acceleration of dilation and constriction) that sophisticated signal processing can separate cognitive from PLR caused pupil size changes( 7 ). Schwalm et al. later used this method to distinguish between mental workload levels of drivers in a simulator( 2 ). In the context of driving, the study of pupillary dilation has mostly focused on mental workload( 8, 9 ).

Driving is generally considered a foveated task (i.e., an object generally needs to be fixated by the driver in order to be perceived). However, drivers can perceive certain potential hazards without or shortly before an explicit fixation. On the other hand, several studies have empirically shown that the mere fixation of a specific object does not imply its perception not its interpretation as hazardous by the driver. For example, in ( 10 ) hazard fixation was found to be unreliable for predicting hazard perception, as an object can either be ‘cognitively overlooked’ or incorrectly judged as non-hazardous by the driver. Thus, if we want to infer information on hazard perception in a driving scene, the fixation-based information is not sufficient. Other physiological signals, such as electrocardiography (ECG) or Galvanic Skin Response (GSR), can help us to disambiguate. However, they usually show a variable and relatively long delay (within several seconds) so that they are not applicable to a realtime use case, e.g. to trigger assistance systems, nor to determine the exact moment in time when a hazard is perceived. Contrary to these physiological parameters, pupil response happens almost instantly and spans only about 2 seconds( 11 ). This lack of a delay allows for a timely interaction with in-vehicle systems.

In this study, we investigate the pupil dilation in immediate response to a hazard during driving. Our aim is to investigate the predictive quality of the pupillary signal to infer hazard perception. Being able to detect hazard perception of a driver reliably via a change in pupil diameter is interesting for multiple reasons: In Underwood, Ngai & Underwood, 2013 the authors perform a hazard perception task where subjects are to press the space bar once they perceive a hazard. Similar experimental setups are common in studying hazard detection, e.g., in ( 12 ) subjects were to honk upon detection of a pedestrian. We could substitute such artificial manual feedback by a noninvasive measurement of pupil dilation. Furthermore, we could disambiguate other stress signals, such as hazard fixation, heart rate changes or the galvanic skin response by use of the pupil diameter: was an object perceived and judged as hazardous?

For the purpose we can built on insights gained from the analysis of mental workload during driving, as the identification of a stress response shares the common problem of isolating a cognitive pupillary dilation from the PLR. For example in( 13 ), the authors find an increase in pupillary dilation with mental workload that is reliable even under the daylight variations of a natural environment. However, the detection of this effect is only possible through averaging over of a large amount of data and by applying statistical methods. Finding a statistically significant difference in a large collection of data does not imply that a useful classification of individual trials towards a specific mental workload state is possible.

In this context, the Index of cognitive activity is of much interest, as its authors claim that it is almost immune to illumination changes. Therefore, a wavelet transformation filters only those pupil changes that did not originate from ambient illumination changes. By analyzing only certain components of the wavelet-transformed signal, we filter for a specific dilation speed and amplitude( 7 ).

For determining a stress level, increasing mean values of the pupil diameter over time are commonly used( 14 ). This averaging has the advantage of being relatively robust towards momentary pupil diameter changes as caused by rapid illumination changes. Pedrotti et al. used a wavelet transformed pupil diameter in a simulated driving task in order to classify different stress levels of the driver( 15 ). Such a procedure is useful when a gradual change in stress level is expected. However, for our application, we are interested in spontaneous, fast stress events and an average filter would delay the detection of the expected steep and short peaks.

In the following, a filtering and classification cascade for the pupil diameter signal is introduced that can be utilized to classify the perception of hazards during driving in a simulator.

## Methods

### Driving simulator experiment

**Fig. 1.**
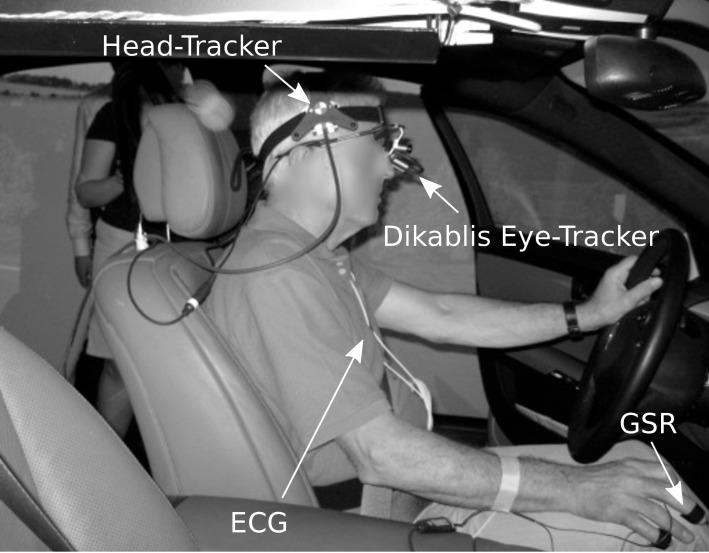
Setup of the vital parameter sensors in the driving simulator.

Thirty-one subjects drove in the moving-base driving simulator ( 16 ) at the Mercedes-Benz Technology Center in Sindelfingen, Germany. The cabin contained a real car body amidst a 360° virtual reality, thus the driving experience was very realistic. Each subject absolved a 40 min drive of 37.5 km length. Nine hazardous situations occurred at predefined positions along the course. A Dikablis essential eye tracker (Ergoneers GmbH, Manching/Germany) recorded eye movements and pupil size at 25 Hz. Simultaneously, we recorded the physiological parameters galvanic skin conductance (GSC, Biotrace+ with finger electrodes) and heart rate (ECG, mobile 3-channel customed EKG). Figure 1 shows the experimental setup. The processing steps required to derive an indicator of hazard perception from these sensors are published in( 10 ).

All subjects were recruited from the department of Neuro-Ophthalmology at the University of Tübingen (Germany). The research study was approved by the Institutional Review Board of the University of Tübingen (Germany) and was performed according to the Declaration of Helsinki. Aim of the original study was to analyze the driving performance of patients with binocular visual field loss (16 patients, 15 control subjects). For the analysis provided here, we do not expect an influence of these groups on the pupil diameter and therefore provide no further interpretation with regard to the visual field defects.

### Pupillary data processing

As we are operating on data recorded in a close to realistic environment, we have to first assure sufficient data quality. In a preprocessing step, we eliminated blinks, partial blinks and unlikely pupil sizes from the data:

The first 30 seconds of the pupil signal were very noisy due to an acclimation phase of the subject in the car. We discarded this relatively short time interval for all subjects. We identified blinks, tracking failures of the eye tracker and pupil size samples that differed by more than 10% from their preceding value (empirically chosen and mainly dependent on pupil detection quality). We eliminated these usually relatively short tracking losses from the data. That produces an artifact spanning up to five samples, given the 25 Hz sampling rate of the eye tracker. Additionally, two samples (corresponding to 40 ms) before and after a blink were removed as well since a partial occlusion of the pupil by a half-closed eyelid may cause the pupil detection to report a smaller size than actual pupil size. To eliminate physiologically unlikely pupil sizes, we used a statistical approach and considered all pupil sizes that exceed the average by more than three standard deviations outliers. Such samples result from a failure of the pupil detection algorithm (e.g., by detecting the iris instead of the pupil). We filled the gaps from missing/eliminated data by a linear interpolation between the neighboring valid samples. This step was necessary as the following frequency-based processing steps require a continuous signal without discontinuities.

**Fig. 2.**
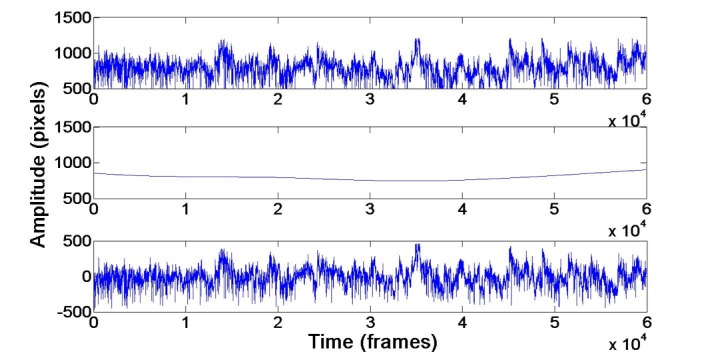
The raw pupil area signal (top) and its reconstruction using the wavelet coefficients (middle). We obtain a detrended signal (bottom) by subtracting the wavelet approximation from the original signal. The sampling rate is 25 frames/second.

Trials with less than 75% of valid data (with interpolated points of the previous step counting towards invalid data) were not included for further analysis. In the next step, we compensate for a non-stationary trend (i.e., a gradual slow change in pupil diameter over several minutes). We identify such a local trend by reconstruction of the original signal from wavelet coefficients that correspond to a low frequency band (see Figure 2). It is necessary to remove such a trend before applying spectral analysis, as it distorts the spectra of the signal at low frequency( 17 ).

**Fig. 3.**
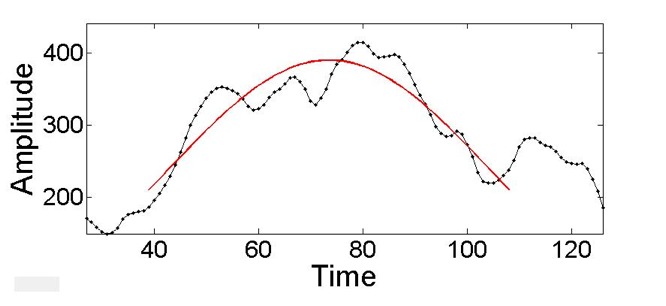
Fit of a parabola to a candidate pupil dilation peak.

A manual analysis of the pupil diameter signal after filtering and smoothing indicated that peaks do indeed occur at the hazardous situations, but also that a simple threshold approach is insufficient to detect them reliably amongst the high noise level. Spurious pupil diameter peaks need to be distinguished from the peaks corresponding to hazardous situations. We employ the method introduced in ( 18 ) for this purpose:

First, we detect zero-crossings of the smoothed first derivative of the pupil diameter signal. They correspond to extrema in the original signal. We consider them as candidate peaks, if their amplitude exceeds 1.5 standard deviations. Then, a parabola is fit to the set of points within a 2.5 second time-window around the peak by least squares quadratic fit (Figure 3) using the *full width at half maximum method*( 19 ). The pupil response to visual detection is supposed to last for 2-2.5 seconds( 11 ), motivating this choice of window width.

### Wavelet Analysis

For each drive, we identified and labelled all hazardous events and the corresponding pupil signal. We automated this process as the driving simulation provided the position of the vehicle on the track and we knew about the position of the pre-programmed hazardous events.

Several different events resulted in a stress or emotional response on different levels of intensity during the driving session. As the illumination within the simulator environment does not change as rapidly and intensely as during actual on-road driving, we can expect these events to have a major impact on pupil dilation. A stress response results in rapid pupil dilation, but also in the following gradual return to normal size. This gradual return is often of oscillatory nature and contains several (decreasing) waves. The more significant the event, the longer this return phase( 20 ). In order to discriminate between possible causes for a pupil dilation, we perform a scale analysis of the time series: wavelet analysis.

**Fig. 4.**
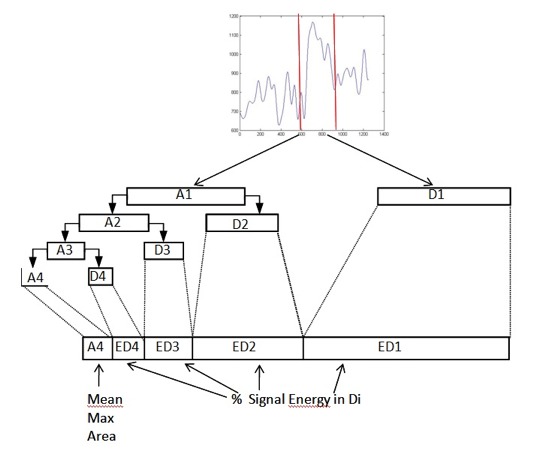
Construction of the feature vector for one candidate peak. Mean, amplitude and area are calculated from the A4 component, the relative energy from the detail coefficients.

The wavelet transform decomposes a signal into wavelets (i.e., small waves with their energy concentrated in time). These wavelets are scaled and shifted copies of a main pattern, called the mother wavelet. In a multiresolution representation, the signal is decomposed into increasingly finer details based on wavelet and scaling functions, which correspond to a high pass and a low pass filter. Precise time information is contained at high frequencies and frequency information at low frequencies. These filters are applied successively to the signal joined by a down sampling by factor 2 (Figure 4). The maximum level of decomposition depends on the relevant time scale of events under consideration( 21 ),( 22 ).

We can separate events at different levels on the arousal scale by partial reconstruction of the signal in only one specific frequency sub-band, which corresponds to the respective arousal level.

For our purpose, we chose to decompose and reconstruct the signal accurately at a time scale of 1-2 s. The pupil can react to stimuli within 200-350 ms and reaches peak response between 500-1000 ms( 11 ). For the 25Hz sampling rate of our eye tracker, this corresponds to the fourth level decomposition.

It is important to select a wavelet that matches the shape and frequency characteristics of the signal we want to separate. The Daubechies wavelet family is optimal in the sense that most of the wavelet coefficients are small or zero, making them well suited for matching smooth polynomial features in a given signal( 23 ).

### Feature extraction

For each of the candidate peaks extracted in the previous step, we applied a temporal window to extract the signal within 1.5 seconds before/after the peak. We then decomposed the signal within this window into sub-band frequencies by means of a discrete wavelet transform with Daubechies 4 (db4) wavelets up to level 4 and extracted the detail and approximation coefficients. From these coefficients we calculated the relative energy of the wavelet, which characterizes the signal’s energy distribution at diff erent frequency bands.

### Classification of pupil size peaks

To discriminate between peaks that occur as an effect of noise and ambient illumination change during the drive from pupil responses to hazardous events, we used a support vector machine (SVM) with radial basis function (RBF) kernel. The feature vectors used for the training of the SVM were composed of the following: amplitude, mean diameter, area of the approximation coefficient A4, and the wavelet relative energy corresponding to the detail coefficients D1-D4 (Figure 4). The SVM selects those criteria and their interactions that help us to distinguish between different kinds of peaks. Such a machine learning approach is sensitive to unbalanced data. In our case, the relatively large amount of peaks occurring during normal driving (that we want to classify as noise) would result in a relatively high classification accuracy, even if the SVM would simply classify every as noise. It would simply neglect the few hazardous events. Therefore, we balanced the number of feature vectors for each class by oversampling of the minority class (i.e., the hazardous events). We trained and tested the SVM using leave-one-out cross-validation and evaluated the classification accuracy separately for each subject by using only training data from the other subjects. This evaluation procedure is almost unbiased and gives a good indication of the cross-subject generalization performance ( 24 ) while it makes good use of our limited training and test data. It should however be noted that the selection of candidate peaks and the construction of the feature vector involves subject-specific adaptation such as the subject’s average pupil diameter and its distribution.

## Results

**Fig. 5.**
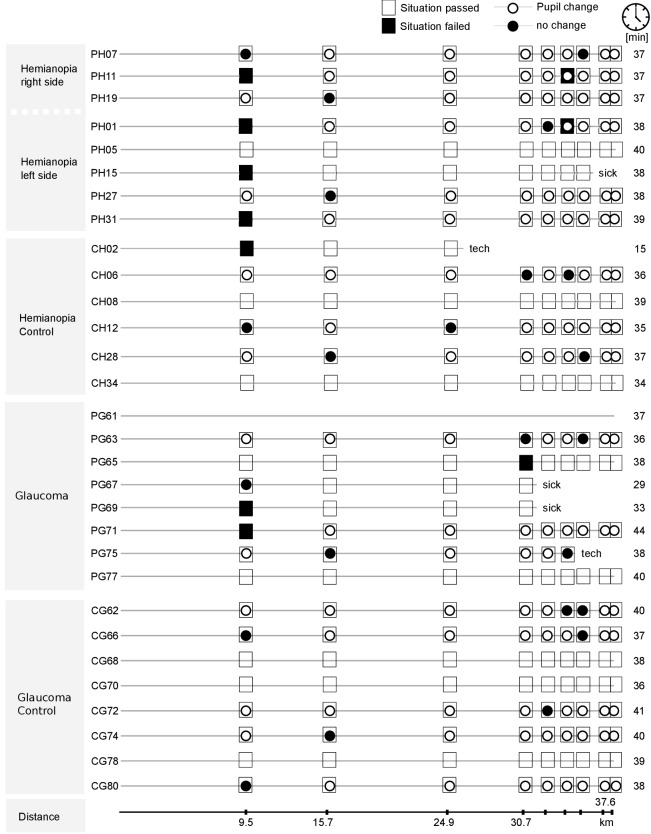
Presence of a detected pupil diameter change at hazardous situations for all subjects and situations. Each row corresponds to the drive of one subject. The squares along the drive correspond to the hazardous situations and are filled black, if the driving instructor judged an inadequate driving response. The inscribed circle shows whether a pupil response was observed (filled white) or not (filled black). Lines without pupil markers correspond to trials excluded from data analysis due to a bad tracking rate of the eye-tracker. In addition, we provide locations of dangerous situations along the route. In each case, where a subject aborted the experiment, the reason (either technical difficulties or motion sickness) is provided.

Figure 5 shows the detailed results of the classification for each subject. A white circle indicates a change in pupil diameter that the classificatory judged relevant; a black circle indicates that such a change was not detected. The surrounding square indicates whether the driving instructor judged the driving response as adequate or not. Both markers have to be considered in conjunction. For example, a black square and black circle indicate a situation that the driver did not perceive and, consequently, did not react to. A white square with a white circle would correspond to a hazard that the subject responded to adequately and that caused a pupil dilation.

**Fig. 6.**
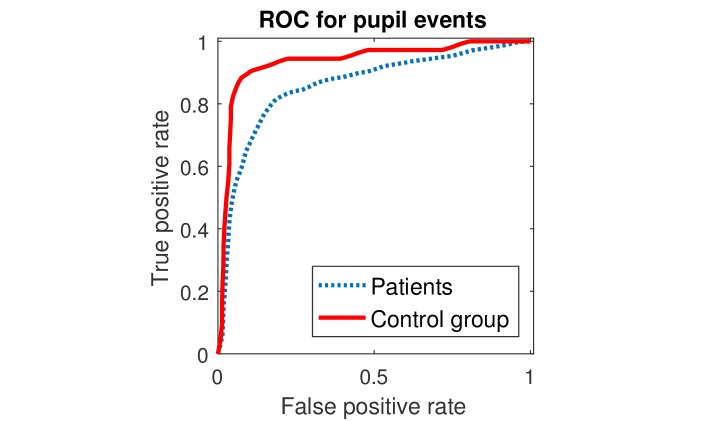
Receiver operating curves of the classification performance for both the visual field defect patients and the control group.

Figure 6 shows the ROC curves for the classification, separately for the patient and the control group. As there were very few inadequate driving responses in the control group, we can expect the curves to differ even in the case that the visual field defect does not have any effect on the pupil diameter. 

Such a prediction assumes that a hazard to which the driver reacted was perceived and via versa a hazard that was not reacted to adequately was overlooked by the driver. From previous analyses of vital parameter data we know that this was not always the case, e.g., some drivers responded inadequately to a hazard they had perceived. Therefore, we cannot expect a perfectly reliable classification result. For our analysis, we decided to predict as many of the hazardous situations from the pupil data as possible and allowed for a moderate number of false positives (so we judge in favor of hazard perception in case of doubt). The numerical classification results are provided in Table 1.

**Table 1.** Results for the prediction of hazardous event perception from the pupil diameter.

	**Control group**	**Patients**
Specificity	0.89	0.78
Sensitivity	0.92	0.83
Precision	0.93	0.87

When we analyze those situations that lead to a failure of the driving test, we can now distinguish between a perceptual failure and a behavioral failure: Subject PH11 fails the first situation without perceiving the hazard. The same subject also fails the sixth situation, but this time perceived the hazard, as a pupil diameter change happens. This might be due to a general awareness of a dangerous situation without knowledge about the exact location. Just as interesting is that we can also derive that PH07 showed an adequate driving behavior to the first and seventh situation, even though the hazardous object was likely not perceived. Being able to include such events in the evaluation of driving performance will allow us to better judge driving safety also for subjects with a more defensive driving style that would require extensive testing before the perceptional deficit becomes obvious in terms of a driving test failure.

Table 2 gives some insights to the false positives that influence the ROC curves and classification results. We can observe that the classification step performs well in filtering only few events from many candidates (e.g., from 86 to 7 for subject CG66). It returns an average of 8±8 false positives, i.e. it classified pupil size peaks as a stress response to a hazard that were not associated with one of the predefined hazardous situations. Without the classifier, an average of 50 false peaks per drive would be reported. For the task at hand we aimed at predicting hazard perception at the predefined hazardous situations. It is possible that some subjects were very careful at several other situations along the route that looked like potential hazards, and therefore, showed valid additional stress responses that we are (wrongly) counting as false positives. In order to decide in favor of hazard perception we accepted a relatively high number of false positives along the complete drive..

**Table 2.** Pupil dilation event classification. F = the number of candidate peaks that did not correspond to a hazard situation but were misclassified as such an event (false positives); D = the number of candidate peaks before the classification step. Subject descriptors indicate the patient groups of hemianopia (PH) and glaucoma (PG) as well as their respective control groups (CH/CG)

Subj.	F	D			Subj.	F	D
PH07	14	31			PG69		
PH50	4	46			PG63	36	94
PH11	4	29			PG75	5	24
PH05					PG61		
PH27	2	34			PG65		
PH25	1	6			PG71	7	42
PH15					PG77		
PH01	5	44			CG74	8	
CH06	8	37			CG66	7	86
CH12	6	41			CG62	10	54
CH08					CG78		
CH28	17	75			CG68		
CH20	7	62			CG80	4	55
CH02					CG72	2	4

## Discussion

Hazard perception involves the input of sensory information and subsequent cognitive processing. This processes result in the identification of potentially dangerous traffic situations. Only the combined process of seeing and identifying a hazard will lead to a stress response. We found that pupil dilation can be utilized to disambiguate hazard perception and adequate driving reaction in a simulated driving scenario.

We employed a filtering and classification cascade that is able to identify sudden stress responses from the pupil data. We aimed at correctly detecting as many of the hazardous situations as possible from the pupil diameter only while trying to minimize the false positives. This allows us to determine whether the subject likely perceived a hazard. Due to the number of false positives during the drive it could not be used as a stand-alone detection system for hazardous situations, e.g., to trigger assistance systems. It only indicates the perception of the driver, if such an event has occurred. We designed the hazard situations to be easily overlooked by the driver and to resemble a looming emergency. They are therefore very attention arousing and stress inducing. For less challenging scenarios where the driver can detect hazardous objects earlier and sufficient reaction time is available, no stress signals would be expected. Eye-tracking measures would then be sufficient.

Pupil dilation events were more absent in those situations that were relatively difficult (e.g. 1, 2 and 6, where subjects actually failed the driving test or had only few reaction time available). That indicates that careful, prospective driving behavior may have resulted in a less intense experience of the hazardous situation for some drivers – or that they were simply lucky to have passed the situation. We further found that there is a large individual variation in the number of predicted stress peaks per subject, likely associated with the level of engagement and emotional arousal of the driver during the test scenario.

We showed that the pupil variation events occur with the detection, recognition and reaction to potentially dangerous events while driving. It indicates the moment at which a potentially dangerous event becomes relevant to awareness. Furthermore, the pupil dynamics can resolve the ambiguity of perception and unexpected uncertainty that plays an important role in detecting and recognizing unexpected dangerous events( 25 ),( 26 ).

As brightness within a simulated world (road surface, sky, vegetation, etc.) varies only about ±5% from the average brightness( 27 ), we can currently not conclude as to whether and to what extend these findings may hold for on-road driving. Yet, the indicator may be useful for studies that require a precise distinction between hazard perception and the behavioral driving response without requiring unnatural behavior such as pressing a button upon detection. The approach may also be used to assess the design of a simulator track as to whether a timely detection of planned hazard scenarios is possible.

## Ethics and Conflict of Interest

The author(s) declare(s) that the contents of the article are in agreement with the ethics described in http://biblio.unibe.ch/portale/elibrary/BOP/jemr/ethics.html and that there is no conflict of interest regarding the publication of this paper.

## Acknowledgements

The authors would like to thank Daimler AG for the possibility to use the moving-base driving simulator for this study.

We would further like to thank Pfizer and MSD Sharp & Dohme GmbH for supporting and enabling this study. The authors declare that there is no conflict of interest regarding the publication of this paper.

Work of the authors is supported by the Institutional Strategy of the University of Tübingen (Deutsche Forschungsgemeinschaft, ZUK 63)

We acknowledge support by Deutsche Forschungsgemeinschaft and Open Access Publishing Fund of University of Tübingen.
